# Hepatitis C virus infection in blood donors from the state of Puebla, Mexico

**DOI:** 10.1186/1743-422X-7-18

**Published:** 2010-01-25

**Authors:** Francisca Sosa-Jurado, Gerardo Santos-López, Belinda Guzmán-Flores, Julia I Ruiz-Conde, Daniel Meléndez-Mena, Martín T Vargas-Maldonado, Ygnacio Martínez-Laguna, Laura Contreras-Mioni, Verónica Vallejo-Ruiz, Julio Reyes-Leyva

**Affiliations:** 1 Banco de Sangre, Hospital de Especialidades, Centro Médico Nacional Manuel Ávila Camacho, Instituto Mexicano del Seguro Social, Puebla, Puebla, Mexico; 2 Laboratorio de Biología Molecular y Virología, Centro de Investigación Biomédica de Oriente, Instituto Mexicano del Seguro Social; Metepec, Puebla, Mexico; 3 Clínica de Hepatitis, Servicio de Gastroenterología, Hospital de Especialidades, Centro Médico Nacional Manuel Ávila Camacho, Instituto Mexicano del Seguro Social, Puebla, Puebla, Mexico; 4 Centro de Investigación en Ciencias Microbiológicas, Instituto de Ciencias, Benemérita Universidad Autónoma de Puebla, Puebla, Puebla, Mexico; 5 Laboratorio Estatal de Salud Pública, Secretaria de Salud del Estado de Puebla, Puebla, Puebla, Mexico

## Abstract

**Background:**

Worldwide, 130 million persons are estimated to be infected with HCV. Puebla is the Mexican state with the highest mortality due to hepatic cirrhosis. Therefore, it is imperative to obtain epidemiological data on HCV infection in asymptomatic people of this region. The objective of present study was to analyze the prevalence of antibodies and genotypes of hepatitis C virus (HCV) in blood donors from Puebla, Mexico.

**Results:**

The overall prevalence was 0.84% (515/61553). Distribution by region was: North, 0.86% (54/6270); Southeast, 1.04% (75/7197); Southwest, 0.93% (36/3852); and Central, 0.79% (350/44234). Ninety-six donors were enrolled for detection and genotyping of virus, from which 37 (38.5%) were HCV-RNA positive. Detected subtypes were: 1a (40.5%), 1b (27.0%), mixed 1a/1b (18.9%), undetermined genotype 1 (5.4%), 2a (2.7%), 2b (2.7%), and mixed 1a/2a (2.7%). All recovered donors with S/CO > 39 were HCV-RNA positive (11/11) and presented elevated ALT; in donors with S/CO < 39 HCV-RNA, positivity was of 30.4%; and 70% had normal values of ALT. The main risk factors associated with HCV infection were blood transfusion and surgery.

**Conclusions:**

HCV prevalence of donors in Puebla is similar to other Mexican states. The most prevalent genotype is 1, of which subtype 1a is the most frequent.

## Background

Hepatitis C virus (HCV) infection is an important public health concern. Worldwide, 130 million persons (prevalence of 2-2.2%) are estimated to be infected. The primary diseases associated with HCV are chronic hepatitis, cirrhosis, and cellular hepatocarcinoma [1-3].

The actual prevalence of HCV is difficult to assess because serological tests do not discriminate among acute, chronic, or resolved infection, and the analyzed groups in most countries are not representative of the general population, such as blood donors, drug users, or individuals with high-risk sexual practices [3,4].

HCV prevalence in Mexico has been analyzed in several studies, reporting an average of 1.4% in the open population and 35% in patients with active hepatitis [5]. Because HCV is relatively variable, it is currently grouped in six genotypes and several subtypes. In Mexico, the prevalence of genotype 1 ranges from 30 to 87.5%, with a predominance of subtypes 1b and 1a. Genotypes 2 and 3 are less frequent and genotypes 4-6 are unusual in Mexican subjects [6].

In Mexico, cirrhosis has shown an increasing tendency, rising from 12,058 cases in 2005 to 12,996 cases in 2006. In addition, cirrhosis is the second cause of death in the 15- to 64-year-old age group, being three times higher in men than in women. Puebla is the Mexican state with the highest mortality due to hepatic cirrhosis [7]. Therefore, it is imperative to obtain epidemiological data on the asymptomatic people, which may contribute to know, in part, the possible causes of the high incidence of hepatic diseases in this region.

The aim of this study was to know the epidemiological profile of relatively recent infected HCV individuals, detected as asymptomatic carriers and who were identified as blood donors in the State of Puebla, Mexico.

## Results

### Seroprevalence of HCV

**Figure 1:**
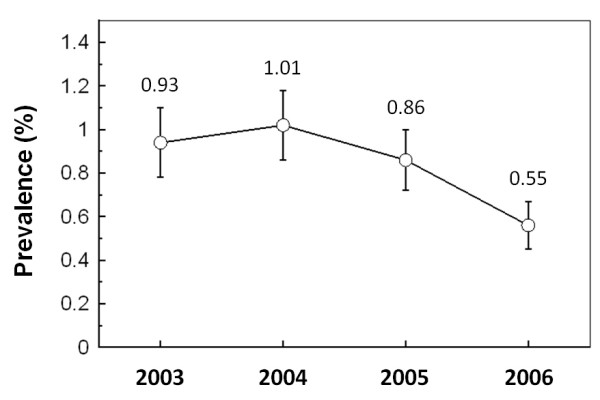
**Prevalence rates of anti-HCV blood donors during the studied period in the state of Puebla**. Specific anti-HCV antibodies were detected using the AxSYM HCV 3.0 assay (Abbott Diagnostics). Bars represent the 95% confidence interval.

**Figure 2:**
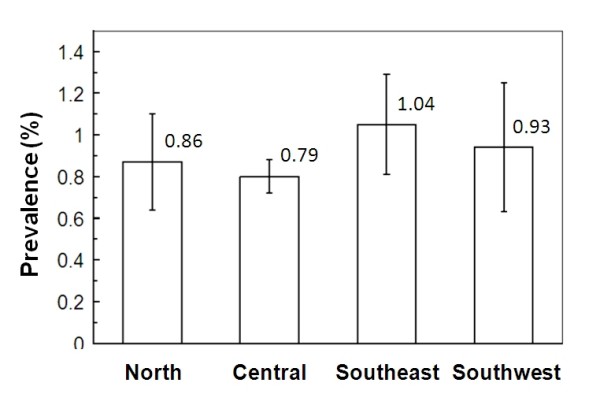
**Distribution of prevalence rates of anti-HCV blood donors by region in the state of Puebla**. The Central Region is known as "Angelopolis". Bars represent the 95% confidence interval.

From 61553 donors analyzed during the study period, 515 (0.84%) were anti-HCV reactive. The overall prevalence descended from 0.93% in 2003 to 0.55% in 2006 (Figure 1). The mean prevalence at the more urbanized central (Angelopolis) region was 0.79% which includes the city of Puebla de los Angeles, the capital of the state. In the other regions, either suburban or rural, prevalence values ranged from 0.86 to 1.04% (Figure 2). No significant difference was observed among these regions (Student's t-test, *p *> 0.05).

**Figure 3:**
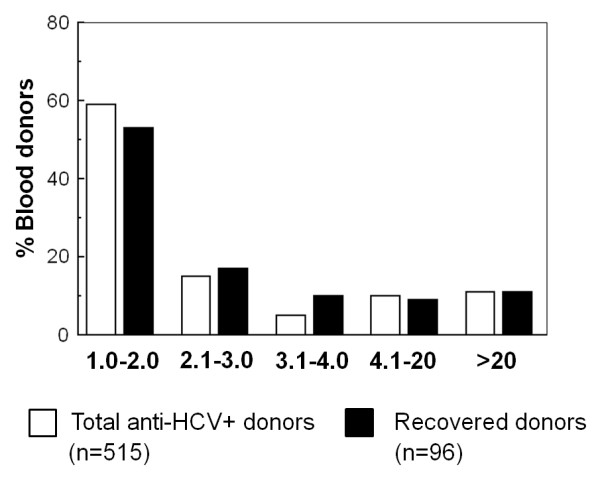
**Distribution of donors (%) according to anti-HCV (S/CO) values**. Comparison of total versus recovered anti-HCV (+) donors.

Low S/CO values (from 1.0 to 4.0) were found in 79% of reactive donors. The rest of the donors presented S/CO from 4.1 to 168, which can be considered from medium to relatively high (Figure 3).

Of 515 reactive donors, 96 accepted to be included in the subsequent studies ("recovered" donors) and were interrogated and sampled from June 2005 and December 2006. From these 96 donors, 80% presented low S/CO values (from 1.0 to 4.0) and the rest presented S/CO from 4.1 to 168 (Figure 3). These data show that this sample of 96 donors is representative of the total population of donors previously analyzed.

### HCV-RNA detection

**Figure 4:**
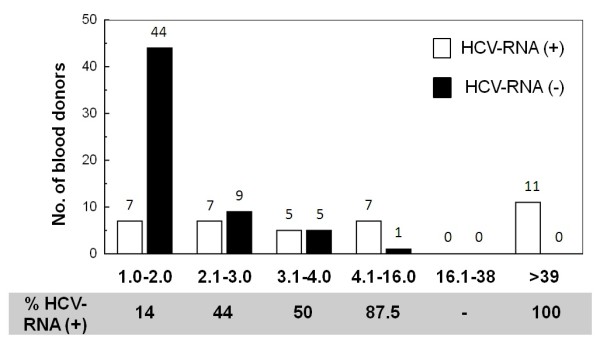
**HCV-RNA positivity in recovered blood donors grouped by anti-HCV S/CO ratio values**.

Of the 96 recovered donors, 37 were HCV-RNA positive (38.5%). The anti-HCV S/CO ratio was related with positivity to HCV-RNA detection; 100% of donors with S/CO > 39 were HCV-RNA positive, whereas donors with lower S/CO values presented progressively reducing percentages of viral RNA detection. In donors with S/CO from 1 to 2, HCV-RNA was detected only in 13.7% (Figure 4).

### Distribution of genotypes

HCV genotypes and subtypes were analyzed in all 37 HCV-RNA positive recovered blood donors. 34 (91.8%) donors had HCV genotype 1. Of these donors, 2 (5.4%), 15 (40.5%), 10 (27.0%), and 7 (18.9%) had subtypes 1 undetermined, 1a, 1b, and 1a/1b, respectively. One donor had genotype 2a (2.7%) and another had subtype 2b (2.7%). One donor (2.7%) presented the mixed 1a/2a genotypes (Table 1).

**Table 1:** Genotype and subtype of HCV in the 37 HCV-RNA-positive recovered donors

Genotype/subtype	Frequency	Percentage (%)
1*	2	5.4
1a	15	40.5
1b	10	27.0
1a/1b	7	18.9
**Genotype 1**	**34**	**91.8**
2a	1	2.70
2b	1	2.70
**Genotype 2**	**2**	**5.40**
1a/2a	1	2.70
**Total**	**37**	**100**

*Undetermined subtype.

### Risk factors

The main risk factors recognized in recovered donors were histories of surgery (29.1%) and of blood transfusion (6.2%), with 46 and 50% of HCV-RNA positive, respectively. Other important risk factors identified were history of migration (15.6%), dental treatments (14.5%), multiple sex partners (5.2%), and living with infected family members (4.16%). In 40.6% of the recovered donors the risk factor could not be identified.

### ALT levels in recovered blood donors

Of 37 HCV-RNA-positive donors, 11 (29.7%) presented significantly high levels of ALT (68.9 ± 7.1 IU/l). All donors HCV-RNA-negative presented normal levels of ALT (30.6 ± 5.6 IU/l), which included individuals with anti-HCV S/CO from 1 to 16.

### Age range of blood donors

The mean age of the 515 reactive donors was 34.5 years, while that of the 96 recovered donors was 34.6. Among recovered donors, positive and negative HCV-RNA, there was no significant difference (34.7 and 38.1 years, respectively; Student's t-test, *p *= 0.234). On the other hand, the mean age of recovered HCV-RNA-positive donors was different between the individuals with anti-HCV S/CO ratios ranging from 1 to 16, and those with S/CO > 39 (35.5; 95% CI, 31.1-39.8 years and 43.3; 95% CI, 38.4-48.3 years, respectively; Student's t-test, *p *= 0.044).

## Discussion

Blood donors are a low-risk population, usually presenting a lower prevalence than the open population. The overall prevalence obtained in the present work was 0.84%, this result is in agreement with other studies on the prevalence among donors in Mexico, which ranges from 0 to 2%, but most of studies report values > 1% [6,8]. It is noteworthy that the prevalence values are descending since 2003 (Figure 1), probably due to the application of a more strict questionnaire to potential donors, as observed previously [9].

An essential item of the epidemiological profile of this type of studies are the risk factors that must be considered during the screening of blood donors in the questionnaire. In contrast to most studies on Mexican populations [6,8], we registered as main risk factor the history of surgery with no prior history of blood transfusion, which was recognized in 29.1% of the anti-HCV positive recovered blood donors. Other studies in Puerto Rico [10] and India [11] have also reported surgery as a first-order risk factor.

Another important parameter is the antibody quantity, estimated by the S/CO ratio values in EIA. S/CO ratios ranged from 1 to 128, and 80% of donors presented low S/CO values (1-4.1), which represent false positive in the majority of cases. All donors were asymptomatic at the moment of sampling, the mean age of blood donors with S/CO ranging from 1 to 4.1 was 35.5 years, whereas in the donors with S/CO > 39 it was 43.3 years. It is noteworthy that the mean age of Mexican infected patients with clinical symptoms has been calculated in 44 years [12], whereas that of Mexican patients with cirrhosis is 52 years [13].

On the other hand, donors with S/CO > 39 presented significantly higher ALT values than donors with low S/CO, which has been observed also in other studies [14-16]; this could be related with a potential hepatic damage.

The HCV-RNA detection is one of the criteria to start therapy that depends also on genotype, viral load, and the degree of liver damage. However, of the 96 recovered donors, only 37 (38.5%) were positive to HCV genome detection, and more than 60% of the anti-HCV positive individuals had no viremia. The presence of specific antibodies against HCV and absence of HCV-RNA is a common finding and may be related with one of the following causes: a) the patient has resolved the infection eliminating the virus, b) the infection is so recent and there is no sufficient viral load to detect the virus and the patient should be continuously monitored, or c) there is a cross reaction with antibodies different from anti-HCV [17-21].

## Conclusions

HCV prevalence among donors in the state of Puebla is similar to that of other Mexican states. The most prevalent genotype was 1 (91.7%) and the most frequent subtype was 1a (40.5%). The main risk factors recognized in recovered donors were histories of surgery and of blood transfusion.

## Methods

### Blood donors

The study population consisted of individuals who donated blood between January 2003 and December 2006 from the State of Puebla, Mexico, at National Health Centre "Manuel Avila Camacho". During this period, 61553 first time donors were analyzed, and were subjected to routine studies by the Blood Bank, according to Mexican Official Standard, NOM-003-SSA2-1993, including a third generation EIA anti-HCV test. After that, we invited the positive anti-HCV donors to participate in this study that included HCV-RNA detection and quantification of alanine-aminotransferase (ALT) activity. Individuals who agreed to enter the study were classified as "recovered" donors. A questionnaire, requesting information on transfusion, surgical or dialysis history, piercing and tattooing, or sexual practices and other data, was applied to recovered donors.

### Detection of anti-HCV

Screening of blood donors was performed using the AxSYM HCV 3.0 assay (Abbott Diagnostics, Wiesbaden, Germany). The AxSYM HCV 3.0 is an indirect microparticle enzyme immunoassay (MEIA) that detects human antibodies against recombinant HCV proteins core, NS3, NS4, and NS5. The values of antibody detection are expressed as the ratio between the signals detected in the sample and the cutoff value (S/CO); samples with S/CO values ≥ 1.0 were considered reactive [22]. All reactive samples were submitted to a second anti-HCV detection test.

### HCV RNA detection

RNA in serum samples was extracted using QIAamp RNA Viral Mini Kit (Qiagen, Valencia, CA). HCV-RNA was amplified using a nested RT-PCR with the two sets of primers corresponding to the 5' UTR, either in the first or second round, as reported previously [23-25]. Reverse transcription and the first round of PCR (25 μl reactions) were performed using One-Step RT-PCR with Platinum Taq (Invitrogen, Carlsbad, CA). Program parameters were 55°C for 30 min (RT), 94°C for 2 min, and 40 cycles of 94°C for 30 sec, 56°C for 30 sec, and 72°C for 30 sec. The second round of PCR (25 μl reactions) was performed using BioMix (Bioline, London, UK) and 2 μl of first-round PCR product. Thermal cycler conditions were 94°C for 2 min and 40 cycles of 94°C for 30 sec, 60°C for 30 sec, and 72°C for 30 sec. Nested RT-PCR was performed by duplicate in all samples and reaction products were analyzed by 1% agarose gel electrophoresis. This home-made RT-PCR has a detection limit of 50 IU per ml (unpublished results), which is comparable with commercial test used for qualitative detection of HCV genome [4].

### Genotyping analysis

Viral genotypes were identified by restriction fragment length polymorphism (RFLP) according to published protocols [25]. Briefly, the 251-bp amplicon obtained in nested RT-PCR was digested with enzymes *Mva*I (Fermentas, Glen Burnie, MD) and *Hinf*I (Promega, Madison, WI) in order to discriminate among viral genotypes 1-6. After that, the 251-bp amplicon was digested with either *Bst*UI or *Scr*FI enzymes (New England Biolabs, Ipswich, MA) to identify viral subtypes. Restriction fragments were analyzed by 15% polyacrylamide gel electrophoresis.

### Alanine-aminotransferase (ALT) determinations

ALT levels were measured with the Advia 1200 Chemistry System (Siemens healthcare Diagnostics Inc, Tarrytown, NY) and the normal reference interval were 4-36 IU/l.

## Competing interests

The authors declare that they have no competing interests.

## Authors' contributions

FSJ, GSL, YML, VVR and JRL participated in the study design, molecular tests, in the data analysis and in drafting and discussing the manuscript. BGF, JIRC, MTVM and LCM carried out the serologic tests of blood donors. DMM received the 96 recovered donors in the Clinic of Hepatitis and assessments clinical and laboratory studies and participated in the analysis of clinical-epidemiological data. All authors read and approved the final manuscript.
